# EcoHIV-Infected Mice Show No Signs of Platelet Activation

**DOI:** 10.3390/v16010055

**Published:** 2023-12-29

**Authors:** Hammodah R. Alfar, Dominic Ngima Nthenge-Ngumbau, Kathryn E. Saatman, Sidney W. Whiteheart

**Affiliations:** 1 Department of Molecular and Cellular Biochemistry, College of Medicine, University of Kentucky, Lexington, KY 40506, USA; alfar.hammodah@uky.edu; 2 Department of Physiology, College of Medicine, University of Kentucky, Lexington, KY 40506, USA; dominic.n.n.nthenge@uky.edu (D.N.N.-N.); k.saatman@uky.edu (K.E.S.)

**Keywords:** platelets, EcoHIV, platelets and EcoHIV, platelets and viruses, viruses

## Abstract

Platelets express several surface receptors that could interact with different viruses. To understand the mechanisms of HIV-1′s interaction with platelets, we chose the EcoHIV model. While EcoHIV is an established model for neuroAIDS, its effects on platelets are ill-defined. Our results indicate that EcoHIV behaves differently from HIV-1 and is cleared from circulation after 48 h post-infection. The EcoHIV course of infection resembles an HIV-1 infection under the effects of combined antiretroviral therapy (cART) since infected mice stayed immunocompetent and the virus was readily detected in the spleen. EcoHIV-infected mice failed to become thrombocytopenic and showed no signs of platelet activation. One explanation is that mouse platelets lack the EcoHIV receptor, murine Cationic Amino acid Transporter-1 (mCAT-1). No mCAT-1 was detected on their surface, nor was any mCAT-1 mRNA detected. Thus, mouse platelets would not bind or become activated by EcoHIV. However, impure virus preparations, generated by Polyethylene Glycol (PEG) precipitation, do activate platelets, suggesting that nonspecific PEG-precipitates may contain other platelet activators (e.g., histones and cell debris). Our data do not support the concept that platelets, through general surface proteins such as DC-SIGN or CLEC-2, have a wide recognition for different viruses and suggest that direct platelet/pathogen interactions are receptor/ligand specific.

## 1. Introduction

Aside from their role in hemostasis, platelets are considered first-line vascular guardians that influence both the innate and adaptive immune systems [1,2,3,4,5]. This influence is mediated by either direct or indirect interactions with immune cells [1,2,3,5]. Platelets can interact with bacteria, parasites, and viruses, which leads to platelet activation and the release of chemokines and cytokines that modulate immune cell functions [3]. While this establishes platelets as potential players in the response to pathogens, it remains unclear how platelets can interact with pathogens and how important that is to systemic immune responses. Most systemic viremias (e.g., HIV-1) present with mild thrombocytopenia [6,7,8,9,10]. This can be mediated through several mechanisms such as decreased platelet production, increased platelet activation/consumption, and platelet destruction through the formation of immune complexes [9,11]. Platelets isolated from virally suppressed or unsuppressed HIV-1-infected individuals are hyperactive and have lower granule content, mitochondrial dysfunction, and intrinsic apoptosis pathway activation [7]. Therefore, hyperactivated platelets appear to contribute to the long-term complications, morbidity, and mortality of HIV-1 infection, as they affect both persistent inflammation and occlusive Cardiovascular Diseases (CVDs) [12]. This might explain why people living with HIV-1have a two-fold increased risk for occlusive CVDs [12,13].

Platelet activation markers (granular contents) such as sCD40L and Platelet Factor 4 (PF4) correlate with the viral load and the nadir count of CD4^+^ T-cells in people living with HIV-1 [3]. Furthermore, several reports suggest that platelets exacerbate a viral infection by acting as a viral reservoir [14]. A recent study has shown that human platelets endocytose HIV-1 during the acute phase of HIV-1 infection and can transmit the virus to CD4^+^ T-cells in resting and activated states through the formation of platelet-CD4^+^ T-cell complexes [15]. Transmission can be inhibited or potentiated upon incubation with an antiplatelet drug or via platelet activation with an agonist, respectively [15]. It seems that platelets can serve as a transient reservoir for the virus to hide from the immune cells until it is time to infect other cell types, e.g., monocytes and CD4^+^ T-cells, through the P-Selectin (CD62P) and P-Selectin Glycoprotein Ligand-1 (PSGL-1) interaction [15]. Similarly, platelets have been shown to transfer HIV-1 to macrophages in vitro, an effect inhibited by abciximab (anti-integrin α_IIb/_β_III_) [14]. These observations suggest that platelets promote virus spreading and aid in infecting other cells. These observations were not surprising, as platelets can transfer CXCR4 to CXCR4-null cells and make them susceptible to targeting by HIV-1 [16]. Therefore, there is an urgent need to better understand the platelet’s role in HIV-1 infection.

Early attempts to create small animal models that express the HIV-1 receptor CD4 and the coreceptors CXCR4 and CCR5 have been challenging because they have not recapitulated robust viral replication and disease progression [17]. Attention shifted to developing humanized mouse models using mice with severe combined immunodeficiency (scid) genetic backgrounds [17]. These models generally rely on the injection of human Peripheral Blood Lymphocytes (PBL), followed by HIV-1 infection, and crosses with strains that enhance human tissue engraftment [17]. These models are useful for evaluating antiretroviral drugs, HIV-1 vaccines, and neuropathogenesis; however, they do not recapitulate HIV-1 progression and are difficult to propagate [17]. The EcoHIV model took a different approach, focusing on modifying an infectious virion [18]. It uses the same genetic code as HIV-1, but gp120 (which binds CD4) was replaced with gp80 of the Murine Leukemia Virus (MLV) [18,19]. This replacement makes the viral construct safer, as it only infects rodents [18,19]. In contrast to the aforementioned models, the EcoHIV model does not require complicated surgeries or xenografts and can be applied to many strains [18,19]. Moreover, EcoHIV has been extensively studied and recapitulates many of the facets of HIV-1 infections found in humans, especially neuropathogenesis [20,21]. Though EcoHIV has advantages, there are some potential limitations. Specifically, the use of gp80 as an envelope protein means that the virion can only interact with and infect cells that express the murine cationic amino acid transporter 1 (mCAT-1) such as macrophages, lymphocytes, microglia, and T-cells [18,20,22]. Thus, it is important to fully characterize the course of EcoHIV infections when using this model.

Since 2005, the EcoHIV model has been widely used to study HIV-1 infections, and, more specifically, researchers have been using this model to study neuroAIDS [18,20,22]. However, there is only one report on the effects of EcoHIV on platelets [23]. The authors showed a successful infection of C57BL/6J mice with thrombocytopenia and a circulating virus in the plasma [23]. Evidence of platelet activation was also detected [23]. Building on these results, we examined this model to further understand the role of platelets during an EcoHIV infection. However, despite a successful EcoHIV infection and established latency in the spleen, the circulating virus was rapidly cleared. Additionally, we did not find evidence of platelet activation using several assays. Two explanations for our results were explored: (1) mouse platelets do not express the gp80-receptor, mCAT-1, which is needed for EcoHIV infection; (2) contaminants in Polyethylene Glycol (PEG) precipitated EcoHIV preparations, but removed by centrifugation onto a sucrose cushion, did activate platelets and generate thrombocytopenia when injected into mice, which was the case for Jones et al. [23]. Thus, while EcoHIV is an important model for the neurological defects associated with HIV-1/AIDS, infections with pure virions do not recapitulate the platelet-related pathologies seen in people living with HIV-1/AIDS.

## 2. Methods

### 2.1. Preparation of EcoHIV Virions

EcoHIV/NDK (hereafter referred to as EcoHIV) virions were prepared as described by Alfar et al. [19]. Briefly, Lenti-X™ cells were transfected with 30 µg of the EcoHIV plasmid. The supernatant containing the virions was harvested every 24 h, filtered using a 0.45 µm filter, and either concentrated with 40% Polyethylene Glycol (PEG)-8000 (40% final) precipitation or centrifugated onto a 20% sucrose cushion to produce PEG-EcoHIV and EcoHIV, respectively. The viral preparations were resuspended in PBS. For all experiments, conditioned media from uninfected cells were processed in parallel and used as a negative control. The quality and titer of the preparations were calculated as described by Alfar et al. [19]. For infections, an intact virion titer was calculated based on detergent-releasable and not total p24.

### 2.2. EcoHIV Infection of Mice

The University of Kentucky Institutional Animal Care and Use Committee (IACUC) approved all murine experiments. Male C57BL/6J mice (12 weeks of age) were injected with 10 µg of intact p24 of EcoHIV via retro-orbital injection. Mice were then euthanized either after 1, 2, 7, or 42 days of infection. Blood was collected via cardiac puncture, the spleen was stored in RNAlater at 4 °C until processing, and the brain samples were prepared for immunohistochemical studies. Hematological analysis of whole blood was performed using the IDEXX ProCyte Dx analyzer (IDEXX Laboratories, Westbrook, ME, USA). 

### 2.3. Platelet Preparation

Mice were euthanized by CO_2_ asphyxiation, and blood was collected into 0.38% Sodium citrate (final concentration) via cardiac puncture and diluted 1:1 with PBS pH 7.4 supplemented with 0.2 U/mL of apyrase and 10 ng/mL of prostaglandin I_2_ (PGI_2_). The blood was separated by centrifugation at 237× *g*, and the resulting platelet-rich plasma (PRP) was further centrifuged at 657× *g* to recover the platelets. The platelets were gently resuspended in HEPES Tyrode buffer (pH 6.5, 20 mM of HEPES/KOH, 128 mM of NaCl, 2.8 mM of KCl, 1 mM of MgCl_2_, 5.5 mM of D-glucose, 12 mM of NaHCO_3_, and 0.4 mM of NaH_2_PO_4_), supplemented with 0.2 U/mL of apyrase and 10 ng/mL of PGI_2_, and washed via centrifugation at 657× *g*. The recovered platelets were finally resuspended in HEPES Tyrode buffer (pH 7.4). Platelet counts were measured using a Z2 counter (Beckman Coulter, Inc., Miami, FL, USA), as previously described [2,24,25].

### 2.4. Flow Cytometry

Platelets (5 × 10^7^ platelets/mL), isolated from uninfected and infected mice, were supplemented with 1 mM of CaCl_2_ and either kept resting or activated with 0.05 U/mL of thrombin for 10 min at 37 °C. Platelets were then fixed with 2% PFA for 1 h and stored at 4 °C until the analysis. Platelets were stained with FITC anti-CD62P (P-Selectin) Ab, APC anti-CD107a Ab (Anti-LAMP1), and PE-anti-CD41/CD61 active form (JON/A clone) for 20 min at 37 °C in the dark. For ex vivo platelet activation with EcoHIV, platelets (5 × 10^7^ platelets/mL) were supplemented with 1 mM of CaCl_2_ and incubated with either 1 µg of intact p24 of EcoHIV or with the same volume of concentrated conditioned media as a control for 1 h at 37 °C. The platelets were then fixed and stained with the appropriate antibodies. For the detection of mCAT-1 on the surface of the platelets, the platelets (5 × 10^7^ platelets/mL) were mixed with either APC-mCAT-1 (Biolegend, CA, USA) or its isotype control antibodies for 20 min at 37 °C. The staining of platelets with the APC-isotype control was used to establish the threshold for positive staining with the APC-mCAT-1 antibody. Lenti-X^TM^ 293T and HEK293-mCAT-1 cells (1 × 10^6^ cells/mL) were used as negative and positive controls, respectively, to validate mCAT-1 antibody staining. To measure platelet neutrophil aggregates, blood was diluted 1:1 with PBS and supplemented with 10 ng/mL of PGI_2_. Aliquots of 50 µL of blood were mixed with 2.5 µL of FITC anti-CD41 (platelet marker) and 2.5 µL of APC anti-Ly6G (neutrophil marker) or their isotype controls for 20 min in the dark at 37 °C. As a positive control, samples from the same preparation were activated with convulxin (20 or 50 ng/mL) for 10 min at RT. The samples were fixed, and the red blood cells (RBCs) were lysed with 1 × BD FACS^TM^ Lysing Solution in the dark for 10 min and stored at 4 °C until the analysis. All cytometry was performed on a BD Symphony A3, and data were analyzed with BD FACSDiva 9.1 and FlowJO (v 10.9.0) software as previously described [24,25].

### 2.5. RNA Isolation and RT-qPCR

The RT-qPCR reactions were performed as described previously [19]. For blood RNA isolation, 150 µL of whole blood (undiluted) was added to RNAlater and stored at −80 °C. The blood RNA was isolated according to the manufacturer’s instructions (Mouse RiboPure™-Blood RNA Isolation Kit, Thermofisher Scientific, Waltham, MA, USA). The spleen RNA isolation was described in [19]. Platelet RNA was isolated using the mirVana™ kit (Thermofisher Scientific, Waltham, MA, USA) according to the manufacturer’s instructions. For all of the RT-qPCR reactions, 500 ng of RNA was used. The copy numbers of *gag* and spliced *vif* were calculated using standards with known copy numbers as described previously [19]. The mCAT-1 expression was normalized to GAPDH, and the gene expression was calculated using the comparative ΔΔCT method [22]. RNA isolated from HEK293 and HEK293-mCAT-1 was used as negative and positive controls, respectively.

### 2.6. Immunohistochemistry

Brains were fixed in neutral buffered 10% formalin, then cryoprotected using a neutral buffered 30% sucrose solution, frozen, and cut into 40 µm-thick coronal sections on a sliding microtome (HM400 R, Microm, Thermofisher Scientific). Immunohistochemistry was carried out on free-floating sections following standard immunohistochemistry protocols published previously [26]. Briefly, endogenous peroxidases were quenched using 3% hydrogen peroxide in 50% methanol for 30 min, and nonspecific binding sites were blocked using 5% normal horse serum. Overnight incubation was carried out at 4 °C in a 1:2000 dilution of a primary antibody (Rat anti-mouse CD68, MCA1957-BioRad, CA, USA), followed by 1 h incubation at room temperature in a 1:1000 dilution of a secondary antibody (biotinylated Donkey anti-Rat IgG (712-065-153, Jackson ImmunoResearch, PA, USA). The signals were amplified using an avidin–biotin–enzyme complex (Vector Laboratories, CA, USA), and immunoreactivity was visualized using diaminobenzidine as a chromogen. The omission of a primary antibody served as a negative control. Sections were mounted on slides; a coverslip was applied, and the samples were imaged using a Zeiss AxioScan.Z1 digital slide scanner at 20× magnification.

### 2.7. Other Methods

Circulating virions were measured in platelet-poor plasma (PPP) by ELISA for HIV-1 p24, according to the manufacturer’s instructions (Alliance HIV-1 p24 ANTIGEN ELISA Kit, PerkinElmer, Shelton, CT, USA). PPP was diluted 4-fold with PBS before the analysis. 

Anti-EcoHIV and human anti-Gag antibodies in plasma were detected by ELISA. Wells were coated with 100 ng of recombinant Gag protein (Abcam, Boston, MA, USA), and, after blocking with 1% BSA, a 1:100 dilution of mouse and human plasma was added. After incubation and washing, bound anti-Gag antibodies were detected with goat anti-mouse IgG-coupled to HRP (Invitrogen, Carlsbad, CA, USA) or goat anti-human IgG-coupled to HRP (Invitrogen). 

Virion preparations were analyzed by SDS-PAGE, followed by either Coomassie brilliant blue staining or Western blotting. The total protein concentrations of sucrose-cushion-purified and PEG-precipitated EcoHIV were calculated using the BCA assay, and 2 µg of total proteins per lane was loaded into a 10% gel. Once separated, the proteins were either stained with Coomassie brilliant blue or subjected to Western blotting using a (1:1000) goat anti-HIV-1 p24 antibody from Abcam (ab19961) and an (1:20,000) alkaline phosphatase anti-goat secondary antibody.

Cytokine levels (CCL2, CCL4, IL-1β, IL-6, IL-10, IFN-γ, soluble P-Selectin, and TNF-α) were measured in the PPP using Mouse Luminex^®^ Discovery Assay (R & D systems, Catalog #: LXSAMSM, Minneapolis, MN, USA) and following the manufacturer’s instructions.

### 2.8. Statistical Analysis

For each experiment, statistical significance was determined using either an unpaired t-test, one-way ANOVA, or two-way ANOVA. When applicable, Dunn’s multiple comparison test was used as a post hoc test. The data are presented as mean ± SEM with statistical significance indicated in each figure as * *p* < 0.05, ** *p* < 0.01, *** *p* < 0.001, and **** *p* < 0.0001

## 3. Results 

### 3.1. Active EcoHIV Was Detected in Mouse Splenocytes Weeks after Infection

**Figure 1:**
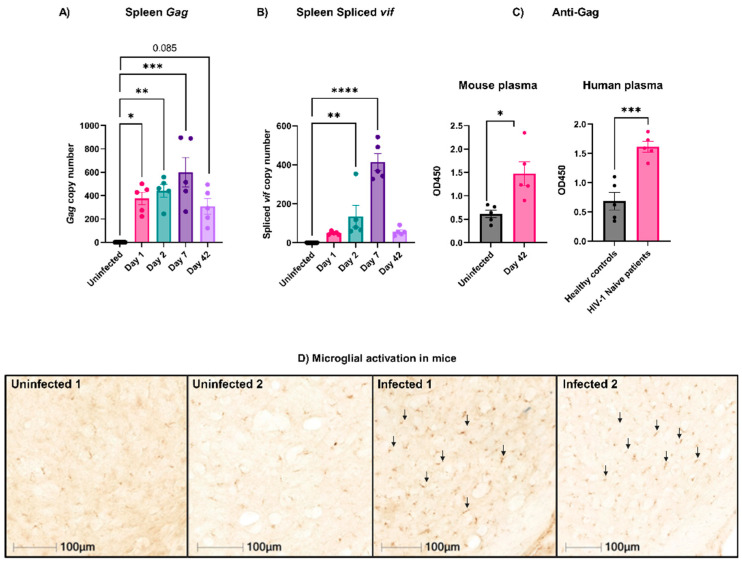
Synthesis of EcoHIV in the spleen peaked after 7 days but then became latent. Mice were infected with EcoHIV (i.v.) and euthanized either after 1, 2, 7, or 42 days. Spleen RNA was isolated, and 500 ng was used for RT-qPCR. (**A**) EcoHIV gag transcript is present in the spleen of infected mice, and its synthesis peaked after 7 days of infection before going into the latent phase on day 42. (**B**) EcoHIV spliced vif is present in the spleen of infected mice, and its synthesis peaked after 7 days of infection before going into the latent phase on day 42. The presence of gag and spliced vif indicates de novo virus synthesis and processing. (**C**) Anti-Gag antibodies in mouse plasma (uninfected and 42 days post infection) (**left** panel). Human plasma samples (healthy controls and HIV-1-naïve patients) were used as negative and positive controls (**right** panel). (**D**) Representative images of uninfected and infected mouse (42 days) brains stained for CD68^+^ microglia. There is a clear activation of the microglial in the brain (black arrows), which is consistent with previously reported data. Each data point represents a mouse. Data graphed as mean ± SEM. Statistical analyses were performed using one-way ANOVA or unpaired *t*-test. * *p* < 0.05 **, *p* < 0.01, *** *p* < 0.001, and **** *p* < 0.0001.

EcoHIV infections in mice have been widely used to model the neurocognitive deficits associated with HIV-1/AIDS [18,20,21,22,27,28,29]. In agreement with many of these studies, we found that following injection, EcoHIV was present in the spleen, peaking on Day 7 post-infection [18,20,22,27]. It was detected as early as Day 1 and persisted for the duration of our study (Day 42; Figure 1A). The activity of EcoHIV in the spleen was evidenced by the presence of the spliced mRNA for the vif protein (Figure 1B). The presence of the spliced mRNA has been used as a metric for de novo virus synthesis that indicates the presence of active and infective viruses in the spleen [18,20,22,27]. Again, the expression peaked on Day 7 but persisted until Day 42. Anti-Gag antibodies were also detected in the infected mouse plasma on Day 42 (Figure 1C, left panel), confirming the generation of an immune response to the virions. To validate the assay, plasma from healthy and HIV-1-naïve patients was used as negative and positive controls, respectively (Figure 1C, right panel). Additionally, the immunohistochemical staining of brains isolated from infected mice on Day 42 revealed an increased expression of CD68 by ~5-fold compared to the uninfected mice, indicating microglia activation at this late stage of infection (Figure 1D). Together, these data validated our preparations for EcoHIV and showed that it recapitulates many of the previously reported properties of this system [18,20,27]. With this system in place, we examined how the EcoHIV infection affected platelets and platelet function.

### 3.2. EcoHIV Was Rapidly Cleared from Circulation

**Figure 2:**
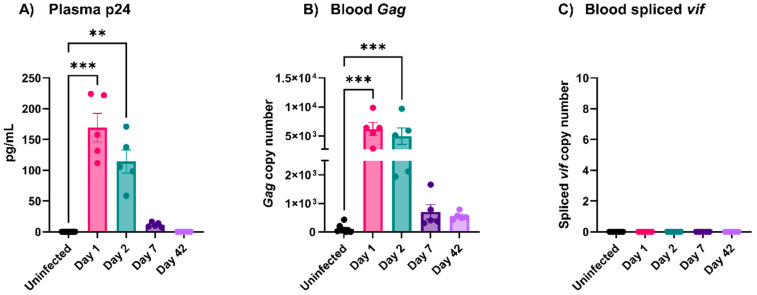
EcoHIV did not continuously circulate. (**A**) The level of p24 protein was measured in the plasma of infected mice on different days. (**B**,**C**) Whole blood RNA (500 ng) was isolated and subjected to RT-qPCR to detect the levels of the EcoHIV transcripts in the blood. Each data point represents a mouse. Data graphed as mean ± SEM. Statistical analyses were performed using one-way ANOVA. ** *p* < 0.01 and *** *p* < 0.001.

HIV-1 is readily detectable in the circulation of people with newly diagnosed HIV-1/AIDS who have not been treated with combined antiretroviral therapy (cART) [30]. To determine if this was true for EcoHIV, we probed plasma and blood samples from mice infected (via *i.v*. injection) with EcoHIV at increasing times. By ELISA, the plasma levels of p24 decreased steadily post-infection and were almost undetectable by Day 7 (Figure 2A). Similarly, the RT-qPCR of whole blood for the presence of *gag* showed a steady decline and was minimal on Days 7 and 42 (Figure 2B). No spliced *vif* was detected in the whole blood at any time point (Figure 2C). Thus, we did not detect active viruses in the blood or plasma of infected mice past Day 7. These data show that, unlike HIV-1, EcoHIV does not persist in circulation and is rapidly cleared, perhaps by the spleen since active virions were detectible there for the duration of our study (Figure 1B). 

### 3.3. EcoHIV-Infected Mice Did Not Have Thrombocytopenia nor Show Signs of Platelet Activation

**Figure 3:**
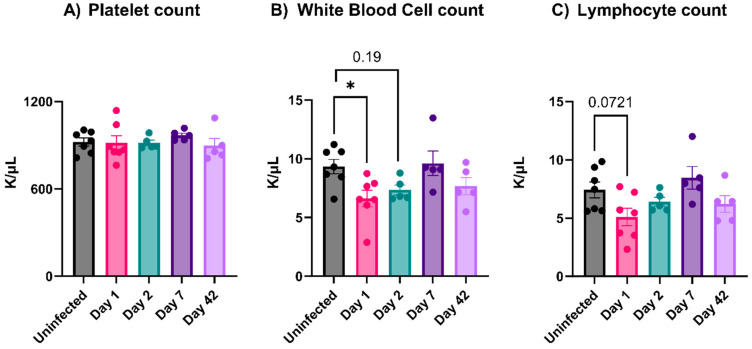
EcoHIV did not induce thrombocytopenia or systemic immune responses in mice. Mice were infected with EcoHIV (i.v.) and euthanized either after 1, 2, 7, or 42 days. (**A**) Infected mice did not develop any degree of thrombocytopenia at any time point. (**B**) Infected mice had a decrease in white blood cell (WBC) count after 24 h, and the effect disappeared over time. (**C**) Infected mice did not develop lymphopenia over time during the infection. Each data point represents a mouse. Data graphed as mean ± SEM. Statistical analyses were performed using one-way ANOVA. * *p* < 0.05.

Mild thrombocytopenia is a well-documented sequela of viral infections in many mammals [11]. Such a decrease in platelet counts during a viral infection can be attributed to either decreased production or enhanced clearance [11]. For people with HIV-1, the thrombocytopenia worsens at advanced disease stages [31,32]. The EcoHIV-infected mice showed no decrease in platelet numbers at any stage post-infection (Figure 3A). There was a slight decrease in white blood cell counts on Day 1, which returned to normal by Day 2 (Figure 3B). Similarly, lymphocyte counts also decreased on Day 1, though not significantly (Figure 3C). Interestingly, the plasma levels of different pro-inflammatory cytokines such as IL-6, IL-10, IL-1β, TNF-α, CCL2, CCL4, soluble P-Selectin, and IFN-γ revealed no increase throughout our study (data not shown). Moreover, none of the infected mice showed overt signs of sickness (e.g., hunched posture, ruffled hair coat, reluctance to move, or decrease in weight; data not shown). Taken together, these data suggest that EcoHIV is different than HIV-1 in promoting mild thrombocytopenia.

**Figure 4:**
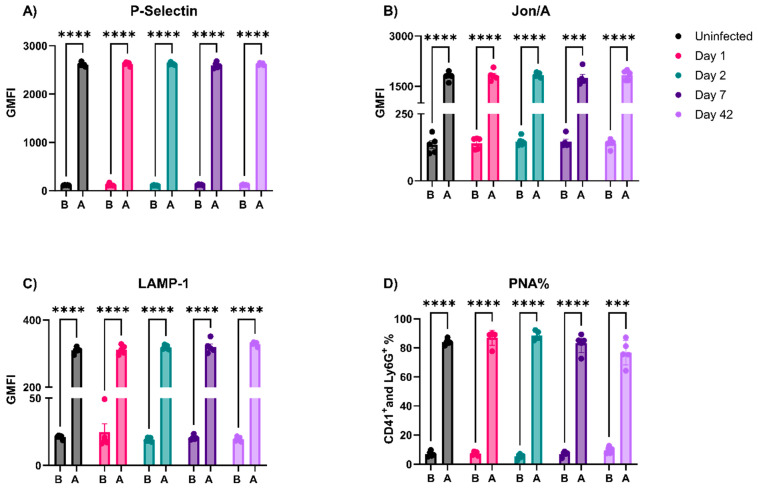
EcoHIV did not activate platelets or induce platelet–neutrophil aggregate (PNA) formation in vivo. Platelets were isolated from mice, fixed with 2% PFA, and stained with (**A**) FITC-P-Selectin, (**B**) PE- Jon/A, and (**C**) APC-LAMP-1 antibodies. No signs of platelet activation were noticed compared to platelets isolated from uninfected mice. To test if the platelets from infected mice were primed, platelets were activated with 0.05 U/mL of thrombin. No differences were noticed between platelets from infected and uninfected mice at baseline or after thrombin activation. (**D**) Whole blood was isolated from infected mice on the indicated day, and the % of platelets (CD41^+^) associated with neutrophils (Ly6G^+^) was calculated on each day. Convulxin (50 ng/mL) was used as a positive control for PNA formation. B: Baseline; A: Activated. Each data point represents a mouse. Data graphed as mean ± SEM. Statistical analyses were performed using two-way ANOVA. *** *p* < 0.001 and **** *p* < 0.0001.

Several viruses interact and appear to activate platelets (e.g., SARS-CoV2, influenza virus, Human Cytomegalovirus (HMCV), and HIV-1) [11,33,34,35,36]. In addition, Mesquita et al. reported sustained platelet activation in people with HIV-1 treated with cART [7]. We probed the status of circulating platelets isolated from EcoHIV-infected mice using cytometry and a battery of surface markers indicative of platelet activation: P-Selectin (α-granule release), LAMP-1 (lysosome release), Jon/A (integrin activation), and the formation of platelet–neutrophil aggregates (PNAs, which require a P-Selectin and PSGL-1 interaction). None of these markers were elevated at any time point, indicating the absence of platelet activation during the course of infection (Figure 4). To confirm the validity of our analysis and to test if the circulating platelets in the infected mice were either hyper- or hypo-activatable, the same platelet preparations were stimulated with a low dose of thrombin (0.05 U/mL). Taken together, platelets from the infected mice are not activated and could be activated with thrombin to the same levels as platelets from the uninfected mice (Figure 4). 

**Figure 5:**
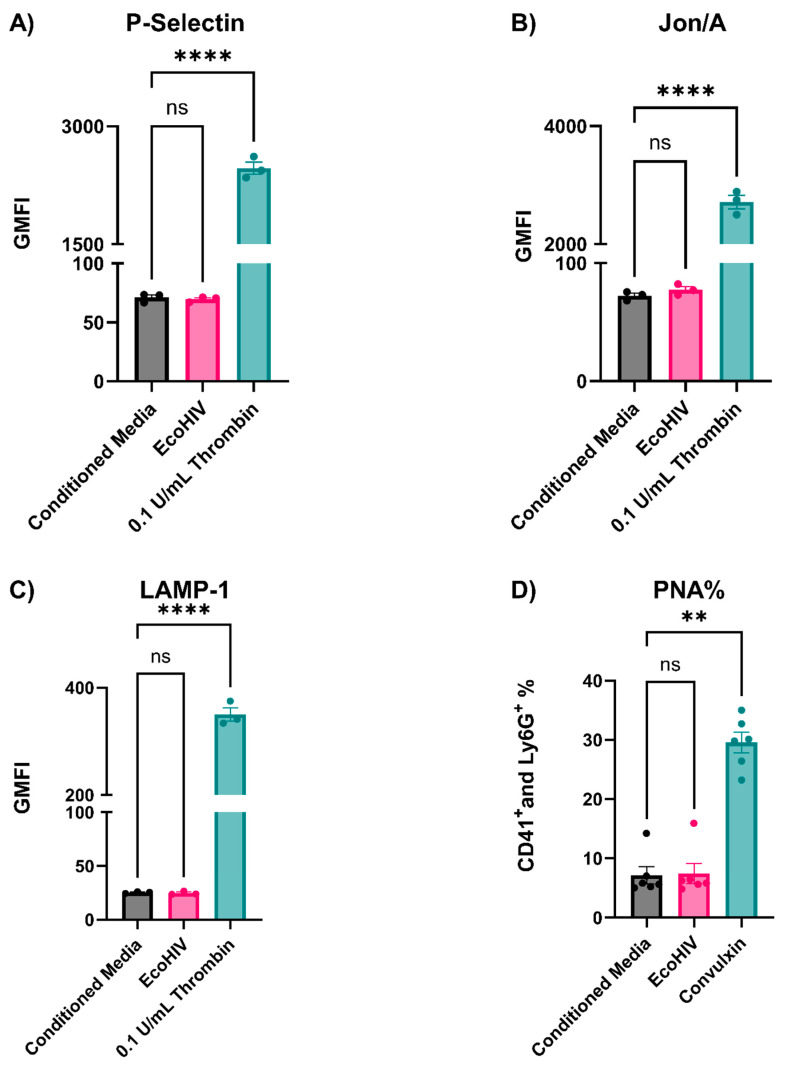
EcoHIV did not activate platelets or induce platelet–neutrophil aggregates (PNA) formation ex vivo. Platelets were isolated from uninfected mice and incubated for 1 h with 1 µg of intact p24 of EcoHIV or equal amounts of conditioned media. They were then fixed with 2% PFA and stained for (**A**) FITC-P-Selectin, (**B**) PE-Jon/A, and (**C**) APC-LAMP-1 antibodies. (**D**) Whole blood was either incubated with conditioned media, 1 µg of intact p24 of EcoHIV, or convulxin (20 ng/mL) as a positive control. B: Baseline; A: Activated. Each data point represents a mouse. Data graphed as mean ± SEM. Statistical analyses were performed using one-way ANOVA. ns: none significant, ** *p* < 0.01 and **** *p* < 0.0001.

These in vivo data were surprising and not consistent with Jones et al., so we sought to determine why EcoHIV fails to affect platelets in mice [23]. First, we incubated and washed platelets with intact EcoHIV and assessed activation by flow cytometry, as shown in Figure 4. Despite clear evidence of activation with defined agonists, thrombin (0.1 U/mL) and convulxin (20 ng/mL), the sucrose-cushion-purified EcoHIV had no detectable effect on P-Selectin (Figure 5A) exposure, Jon/A binding (Figure 5B), or LAMP-1 exposure (Figure 5C). Moreover, the addition of EcoHIV to whole blood failed to cause PNA formation (Figure 5D). These ex vivo results were consistent with Jones et al., as they have shown that blood incubated with EcoHIV does not induce P-Selectin exposure on platelets [23]. These data indicate that EcoHIV does not activate platelets ex vivo.

### 3.4. Mouse Platelets Did Not Express mCAT-1

**Figure 6:**
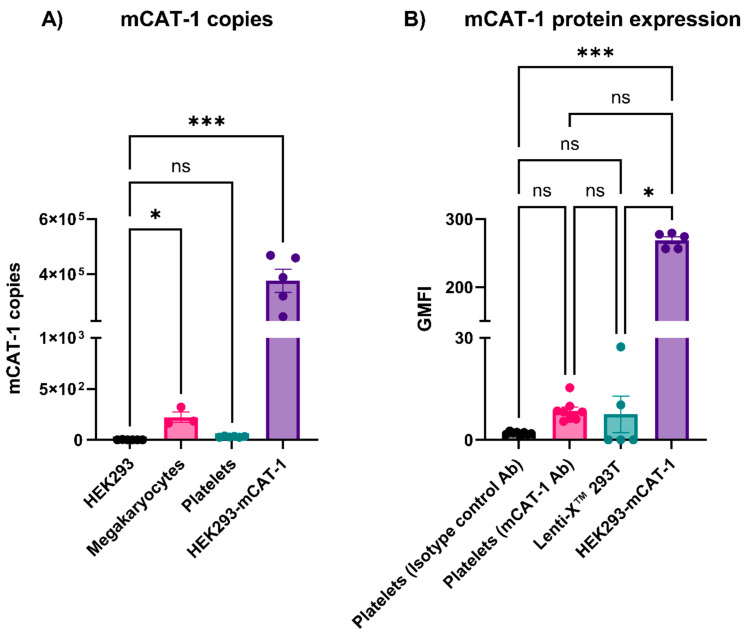
Platelets did not contain mCAT-1 mRNA or express mCAT-1 protein on their surface. (**A**) RNA was isolated from HEK293, megakaryocytes, platelets, and HEK293-mCAT-1 and subjected to RT-qPCR for the detection of mCAT-1 transcripts (500 ng of RNA). (**B**) Platelets were stained with mCAT-1 or its isotype control antibodies. Platelets were identified based on their size and forward/side scatters. Lenti-X^TM^ 293T and HEK293-mCAT-1 were used as negative and positive controls for the specificity of the antibody, respectively. GMFI: Geometric Mean Fluorescence Intensity. Data graphed as mean ± SEM. Statistical analyses were performed using one-way ANOVA. ns: none significant, * *p* < 0.05 and *** *p* < 0.001.

Viruses can interact with platelets and affect coagulation factors, which can increase the risk of thrombus formation [4,5,10,34,35,36,37,38]. Platelets have multiple surface receptors (e.g., DC-SIGN, CLEC-2, and integrins) that can interact with different viruses; however, it is unclear how/if these interactions lead to platelet activation [11]. Virus/receptor signaling could be involved, or the receptors could mediate viral endocytosis and activation through endosomal Toll-like receptors (TLRs such as TLR7 and TLR9) [10,39]. EcoHIV contains gp80 of MLV instead of gp120 of HIV-1 and thus binds to mCAT-1 instead of CD-4 [20]. Since EcoHIV enters cells through mCAT-1, we wondered whether platelets have mCAT-1 transcripts or if they do express mCAT-1 on their surface [20]. By RT-qPCR, mCAT-1 transcripts were undetectable in mouse platelets but were present, at low levels, in their progenitors, megakaryocytes prepared from mouse femurs (Figure 6A). This is consistent with the RNAseq data reported by Zeiler et al. [40]. To confirm the lack of mCAT-1, we also performed a flow cytometry analysis on the platelets using an anti-mCAT-1 antibody and an isotype control. No significant anti-mCAT-1 binding was detected on the platelet surface when compared to two negative controls: an isotype control IgG antibody and Lenti-X^TM^ 293T cells (which do not express mCAT-1) (Figure 6B). As a positive control, HEK293 cells expressing mCAT-1 gave a robust signal in this assay. These data clearly show that mouse platelets lack mCAT-1 and thus would not be expected to bind or respond to EcoHIV.

### 3.5. Preparation Purity Affects How EcoHIV Affects Platelets

**Figure 7:**
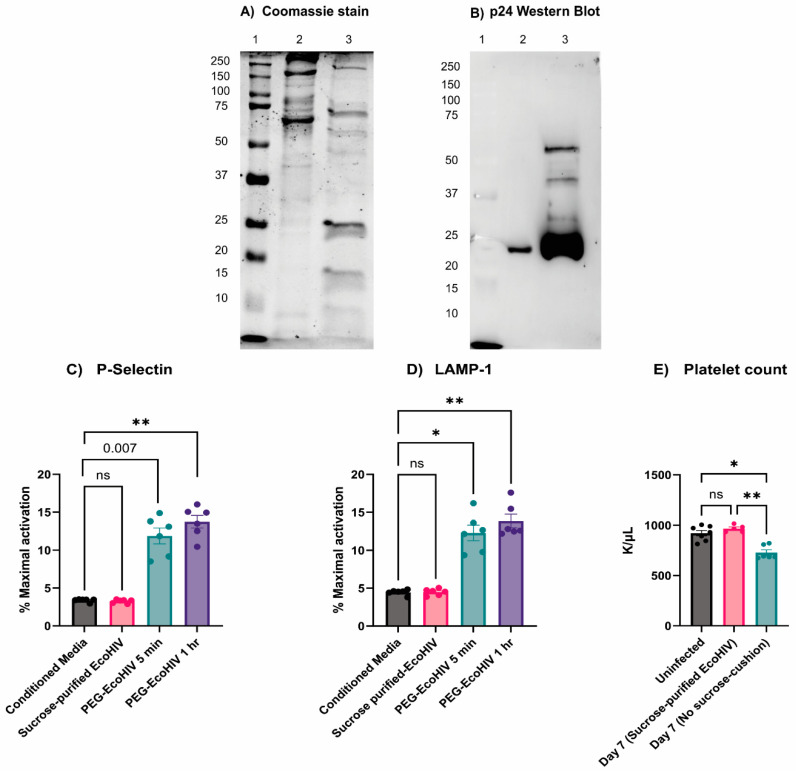
Polyethylene Glycol precipitated EcoHIV (PEG-EcoHIV)-activated platelets ex vivo, but infectious EcoHIV purified on a sucrose cushion did not. (**A**) Coomassie stain of equal amount of total protein (2 µg/lane) of PEG-EcoHIV (lane 2) and sucrose-cushion-purified EcoHIV (lane 3). Lane 1 is a molecular weight ladder. The Coomassie stain reveals how dirty the PEG precipitation is. (**B**) Western blotting of p24 after loading an equal amount of total protein (2 µg/lane) of PEG-EcoHIV (lane 2) and sucrose-cushion-purified EcoHIV (lane 3). The Coomassie stain and the WB of p24 reveal that the specific activity (more p24 with fewer contaminants) of the sucrose-cushion-purified EcoHIV is much higher than that of PEG-EcoHIV. (**C**,**D**) Platelets incubated with sucrose-cushion-purified EcoHIV or its control-conditioned media failed to activate platelets ex vivo, as measured by P-Selectin and LAMP-1 expression. However, platelets incubated with PEG-EcoHIV activated the platelets very rapidly because of the contaminants precipitated during the EcoHIV concentration process. The percent maximal activation was calculated as a percent of 0.1 U/mL of thrombin activation of platelets. (**E**) Platelet count decreased after 7 days of EcoHIV i.v. injection, which was concentrated without the presence of a sucrose cushion and compared to uninfected and sucrose-cushion-purified EcoHIV-infected mice. Each data point represents a mouse. Data graphed as mean ± SEM. Statistical analyses were performed using one-way ANOVA. ns: none significant, * *p* < 0.05 and ** *p* < 0.01.

In our studies, EcoHIV failed to activate platelets, likely due to a lack of the receptor for gp80, mCAT-1. However, others have shown that EcoHIV preparations can activate platelets in vivo but not ex vivo [23]. A potential explanation for this may lie in how virions are purified. There are two general ways to concentrate and purify viral particles from tissue culture media: sedimentation onto a sucrose cushion or precipitation with PEG [19]. The sucrose cushion purification scheme is considered the gold standard method to produce pure viral particles, but the PEG precipitation method is simpler [19]. When we prepared EcoHIV using the two methods, there were significant differences in the quality of the two EcoHIV preparations. In Figure 7A, the Coomassie staining of the preparations showed very different protein patterns, with the PEG-precipitated material having many more detectible bands with high molecular weights. The Western blotting of equal amounts of protein from these two preparations with an anti-p24 antibody further highlighted the differences (Figure 7B). The sucrose-cushion-purified material was much more enriched for p24, suggesting that the PEG-precipitated material was less pure and more contaminated with potentially platelet-activating Damage-Associated Molecular Patterns (DAMPs) and Pathogen-Associated Molecular Patterns (PAMPs) (e.g., DNA, histones, and organelle membranes) not related to EcoHIV. Since our results contradicted those of Jones et al., who prepared their virus by sedimentation of conditioned media from transfected cells without using a sucrose cushion to purify intact virions, we compared three preparations (sucrose-cushion-purified EcoHIV, PEG-EcoHIV, and no-sucrose-cushion EcoHIV) both in vitro and in vivo [23]. As shown above, the sucrose-cushion-purified EcoHIV did not activate platelets, and there was no increase in surface P-Selectin or LAMP-1 expression (Figure 7C,D). However, the PEG-precipitated material did elicit a response in both flow cytometry assays. In vivo, the no-sucrose-cushion EcoHIV caused mild thrombocytopenia on Day 7 post-infection, similar to what has been reported by Jones et al., but the sucrose-cushion-purified material did not (Figure 7E) [23]. This indicates that the no-sucrose-cushion EcoHIV preparations have precipitates that activate platelets and induce their clearance from circulation, but the sucrose-cushion-purified EcoHIV did not. Preparations made from untransfected cells using either method were without effect in any of these assays (Figure 7), and PEG alone did not affect platelet activation (data not shown).

## 4. Discussion

Multiple studies report platelet activation and thrombocytopenia during viral infections [10,33,37,38]. Platelets express several surface receptors, allowing them to interact with viruses; however, how these interactions affect platelet activation is unclear [11,33]. The EcoHIV mouse model appeared to be an ideal system for studying viremia by an HIV-1-like virus. The infected animals showed many of the symptoms of HIV-1/AIDS, especially the effects on the blood–brain barrier and neurocognition [18,20,28]. Jones et al. reported the effects of EcoHIV infections on mouse platelets [23]. However, despite successful infections with detectible viruses in the spleen (Figure 1A,B), anti-EcoHIV antibodies in the plasma (Figure 1C), and activated microglia in the brain (Figure 1D), we failed to detect thrombocytopenia (Figure 3A) or any signs of platelet activation (Figure 4 and Figure 5). However, we did detect signs of platelet activation and thrombocytopenia when impure preparations of EcoHIV were used (Figure 7). Thus, from our results, it appears that EcoHIV does not mimic the platelet-related symptoms of HIV-1/AIDS in humans noted by several groups [7,10,11].

There are at least three potential explanations for why our results differ from those of Jones et al. [23]. First, we used i.v. injections as our route of infection instead of i.p. injections. In circulation, we saw a rapid clearance of virions (Figure 2). It seems possible that interactions with immune cells in the peritoneum vs. those in circulation could account for the differences in the responses to the virions. Intracranial, i.v., and i.p. routes have all been used for EcoHIV infection, but there have been no systematic comparisons of how the route affects the infection course. Second, our data show how impurities in the viral preparations make a difference (Figure 7). Preparations contaminated by cell fragments and other culture debris caused thrombocytopenia and activated platelets. It is well known that DAMPs and PAMPs activate platelets via their TLR receptors [10,11]. Thus, the cell debris rather than the virions could be causing the effects [41]. Jones et al. did not purify their EcoHIV virions on a sucrose cushion, which might explain the effects on platelets they have seen. Finally, we failed to detect mCAT-1, the EcoHIV receptor, on the surface of the mouse platelets (Figure 6B). Mouse platelets do not express Fc receptors and thus cannot react to opsonized viruses (anti-EcoHIV antibodies were generated in the mice; Figure 1D) [42]. Thus, there may not be a specific way for the EcoHIV virions to interact with circulating platelets. Such data argue against our original contention that platelets can globally interact with circulating viruses via broadly specific surface proteins (e.g., CLEC-2, DC-SIGN, and integrins). At least in this system, some receptor-mediated action is required.

In summary, despite being a useful model for the neurocognitive impairment related to long-term infections with HIV-1, EcoHIV did not recapitulate the effects on platelets that have been reported in people living with HIV-1/AIDS [7,10,18,20,21,22,27,28,29]. Our studies highlight the challenges in generating mouse models to probe the effects of viremia on platelets and identify some caveats that should be addressed in future studies.

## Data Availability

This study did not generate a dataset or code. Data are contained within the article.

## References

[1] Semple J.W., Italiano J.E., Freedman J. (2011). Platelets and the immune continuum. Nat. Rev. Immunol..

[2] Holly S.P., Gera N., Wang P., Wilson A., Guan Z., Lin L., Cooley B., Alfar H.R., Patil R.G., Piatt R. (2019). Ether lipid metabolism by AADACL1 regulates platelet function and thrombosis. Blood Adv..

[3] Hottz E.D., Bozza F.A., Bozza P.T. (2018). Platelets in immune response to virus and immunopathology of viral infections. Front. Med..

[4] Joshi S., Smith A.N., Prakhya K.S., Alfar H.R., Lykins J., Zhang M., Pokrovskaya I., Aronova M., Leapman R.D., Storrie B. (2023). Ferric Chloride-Induced Arterial Thrombosis and Sample Collection for 3D Electron Microscopy Analysis. JoVE J. Vis. Exp..

[5] Matharu S.S., Nordmann C.S., Ottman K.R., Akkem R., Palumbo D., Cruz D.R., Campbell K., Sievert G., Sturgill J., Porterfield J.Z. (2023). Deep learning, 3D ultrastructural analysis reveals quantitative differences in platelet and organelle packing in COVID-19/SARSCoV2 patient-derived platelets. Platelets.

[6] Scaradavou A. (2002). HIV-related thrombocytopenia. Blood Rev..

[7] Mesquita E.C., Hottz E.D., Amancio R.T., Carneiro A.B., Palhinha L., Coelho L.E., Grinsztejn B., Zimmerman G.A., Rondina M.T., Weyrich A.S. (2018). Persistent platelet activation and apoptosis in virologically suppressed HIV-infected individuals. Sci. Rep..

[8] Ojha A., Nandi D., Batra H., Singhal R., Annarapu G.K., Bhattacharyya S., Seth T., Dar L., Medigeshi G.R., Vrati S. (2017). Platelet activation determines the severity of thrombocytopenia in dengue infection. Sci. Rep..

[9] Flaujac C., Boukour S., Cramer-Bordé E. (2010). Platelets and viruses: An ambivalent relationship. Cell. Mol. Life Sci..

[10] Banerjee M., Huang Y., Joshi S., Popa G.J., Mendenhall M.D., Wang Q.J., Garvy B.A., Myint T., Whiteheart S.W. (2020). Platelets endocytose viral particles and are activated via TLR (toll-like receptor) signaling. Arterioscler. Thromb. Vasc. Biol..

[11] Assinger A. (2014). Platelets and infection–an emerging role of platelets in viral infection. Front. Immunol..

[12] Freiberg M.S., Chang C.-C.H., Kuller L.H., Skanderson M., Lowy E., Kraemer K.L., Butt A.A., Goetz M.B., Leaf D., Oursler K.A. (2013). HIV infection and the risk of acute myocardial infarction. JAMA Intern. Med..

[13] UNAIDS (2020). Global HIV & AIDS Statistics—2020 Fact Sheet. https://www.unaids.org/en/resources/fact-sheet.

[14] Real F., Capron C., Sennepin A., Arrigucci R., Zhu A., Sannier G., Zheng J., Xu L., Massé J.-M., Greffe S. (2020). Platelets from HIV-infected individuals on antiretroviral drug therapy with poor CD4+ T cell recovery can harbor replication-competent HIV despite viral suppression. Sci. Transl. Med..

[15] Simpson S.R., Singh M.V., Dewhurst S., Schifitto G., Maggirwar S.B. (2020). Platelets function as an acute viral reservoir during HIV-1 infection by harboring virus and T-cell complex formation. Blood Adv..

[16] Rozmyslowicz T., Majka M., Kijowski J., Murphy S.L., Conover D.O., Poncz M., Ratajczak J., Gaulton G.N., Ratajczak M.Z. (2003). Platelet-and megakaryocyte-derived microparticles transfer CXCR4 receptor to CXCR4-null cells and make them susceptible to infection by X4-HIV. Aids.

[17] Hatziioannou T., Evans D.T. (2012). Animal models for HIV/AIDS research. Nat. Rev. Microbiol..

[18] Potash M.J., Chao W., Bentsman G., Paris N., Saini M., Nitkiewicz J., Belem P., Sharer L., Brooks A.I., Volsky D.J. (2005). A mouse model for study of systemic HIV-1 infection, antiviral immune responses, and neuroinvasiveness. Proc. Natl. Acad. Sci. USA.

[19] Alfar H.R., Pariser D.N., Chanzu H., Joshi S., Coenen D.M., Lykins J., Prakhya K.S., Potash M.J., Chao W., Kelschenbach J. (2023). Protocol for optimizing production and quality control of infective EcoHIV virions. STAR Protoc..

[20] Gu C.-J., Borjabad A., Hadas E., Kelschenbach J., Kim B.-H., Chao W., Arancio O., Suh J., Polsky B., McMillan J. (2018). EcoHIV infection of mice establishes latent viral reservoirs in T cells and active viral reservoirs in macrophages that are sufficient for induction of neurocognitive impairment. PLoS Pathog..

[21] Li H., McLaurin K.A., Mactutus C.F., Booze R.M. (2021). A rat model of EcoHIV brain infection. JoVE J. Vis. Exp..

[22] Omeragic A., Kara-Yacoubian N., Kelschenbach J., Sahin C., Cummins C.L., Volsky D.J., Bendayan R. (2019). Peroxisome Proliferator-Activated Receptor-gamma agonists exhibit anti-inflammatory and antiviral effects in an EcoHIV mouse model. Sci. Rep..

[23] Jones L.D., Jackson J.W., Maggirwar S.B. (2016). Modeling HIV-1 induced neuroinflammation in mice: Role of platelets in mediating blood-brain barrier dysfunction. PLoS ONE.

[24] Smith A.N., Joshi S., Chanzu H., Alfar H.R., Shravani Prakhya K., Whiteheart S.W. (2023). α-Synuclein is the major platelet isoform but is dispensable for activation, secretion, and thrombosis. Platelets.

[25] Prakhya K.S., Vekaria H., Coenen D.M., Omali L., Lykins J., Joshi S., Alfar H.R., Wang Q.J., Sullivan P., Whiteheart S.W. (2023). Platelet glycogenolysis is important for energy production and function. Platelets.

[26] Kizhakke Madathil S., Evans H.N., Saatman K.E. (2010). Temporal and regional changes in IGF-1/IGF-1R signaling in the mouse brain after traumatic brain injury. J. Neurotrauma.

[27] Hadas E., Borjabad A., Chao W., Saini M., Ichiyama K., Potash M.J., Volsky D.J. (2007). Testing antiretroviral drug efficacy in conventional mice infected with chimeric HIV-1. Aids.

[28] Bertrand L., Méroth F., Tournebize M., Leda A.R., Sun E., Toborek M. (2019). Targeting the HIV-infected brain to improve ischemic stroke outcome. Nat. Commun..

[29] Li H., McLaurin K.A., Illenberger J.M., Mactutus C.F., Booze R.M. (2021). Microglial HIV-1 expression: Role in HIV-1 associated neurocognitive disorders. Viruses.

[30] Cohen M.S., Shaw G.M., McMichael A.J., Haynes B.F. (2011). Acute HIV-1 infection. N. Engl. J. Med..

[31] Nascimento F.G., Tanaka P.Y. (2012). Thrombocytopenia in HIV-infected patients. Indian J. Hematol. Blood Transfus..

[32] Getawa S., Aynalem M., Bayleyegn B., Adane T. (2021). The global prevalence of thrombocytopenia among HIV-infected adults: A systematic review and meta-analysis. Int. J. Infect. Dis..

[33] Koupenova M., Livada A.C., Morrell C.N. (2022). Platelet and megakaryocyte roles in innate and adaptive immunity. Circ. Res..

[34] Sim M., Alfar H., Hollifield M., Chung D., Fu X., Banerjee M., Li X., Thornton A., Porterfield J., Sturgill J. (2021). HIV-1 and SARS-CoV2 both cause protein s, but through different mechanisms. Res. Pract. Thromb. Haemost..

[35] Sim M., Alfar H., Hollifield M., Chung D.W., Fu X., Banerjee M., Peng C., Li X., Thornton A., Porterfield J.Z. (2022). Unfolded Von Willebrand Factor Interacts with Protein S and Limits Its Anticoagulant Activity. Blood.

[36] Sim M.M., Banerjee M., Hollifield M., Alfar H., Li X., Thornton A., Porterfield J.Z., Sturgill J., Sievert G.A., Barton-Baxter M. (2020). Inflammation drives coagulopathies in SARS-CoV-2 Patients. Blood.

[37] Koupenova M., Corkrey H.A., Vitseva O., Manni G., Pang C.J., Clancy L., Yao C., Rade J., Levy D., Wang J.P. (2019). The role of platelets in mediating a response to human influenza infection. Nat. Commun..

[38] Koupenova M., Vitseva O., MacKay C.R., Beaulieu L.M., Benjamin E.J., Mick E., Kurt-Jones E.A., Ravid K., Freedman J.E. (2014). Platelet-TLR7 mediates host survival and platelet count during viral infection in the absence of platelet-dependent thrombosis. Blood J. Am. Soc. Hematol..

[39] Cognasse F., Hamzeh H., Chavarin P., Acquart S., Genin C., Garraud O. (2005). Evidence of Toll-like receptor molecules on human platelets. Immunol. Cell Biol..

[40] Zeiler M., Moser M., Mann M. (2014). Copy number analysis of the murine platelet proteome spanning the complete abundance range. Mol. Cell. Proteom..

[41] Cognasse F., Nguyen K.A., Damien P., McNicol A., Pozzetto B., Hamzeh-Cognasse H., Garraud O. (2015). The inflammatory role of platelets via their TLRs and Siglec receptors. Front. Immunol..

[42] López J.A. (2013). The platelet Fc receptor: A new role for an old actor. Blood J. Am. Soc. Hematol..

